# A Grouping Differential Evolution Algorithm Boosted by Attraction and Repulsion Strategies for Masi Entropy-Based Multi-Level Image Segmentation

**DOI:** 10.3390/e24010008

**Published:** 2021-12-21

**Authors:** Seyed Jalaleddin Mousavirad, Davood Zabihzadeh, Diego Oliva, Marco Perez-Cisneros, Gerald Schaefer

**Affiliations:** 1 Computer Engineering Department, Hakim Sabzevari University, Sabzevar 96179-76487, Iran; d.zabihzadeh@hsu.ac.ir; 2 Departamento de Innovación Basada en la Información y el Conocimiento, Universidad de Guadalajara, CUCEI, Guadalajara 44430, Mexico; 3 Department of Computer Science, Loughborough University, Loughborough LE11 3TT, UK; gerald.schaefer@ieee.org

**Keywords:** image segmentation, multi-level image thresholding, optimisation, differential evolution, clustering

## Abstract

Masi entropy is a popular criterion employed for identifying appropriate threshold values in image thresholding. However, with an increasing number of thresholds, the efficiency of Masi entropy-based multi-level thresholding algorithms becomes problematic. To overcome this, we propose a novel differential evolution (DE) algorithm as an effective population-based metaheuristic for Masi entropy-based multi-level image thresholding. Our ME-GDEAR algorithm benefits from a grouping strategy to enhance the efficacy of the algorithm for which a clustering algorithm is used to partition the current population. Then, an updating strategy is introduced to include the obtained clusters in the current population. We further improve the algorithm using attraction (towards the best individual) and repulsion (from random individuals) strategies. Extensive experiments on a set of benchmark images convincingly show ME-GDEAR to give excellent image thresholding performance, outperforming other metaheuristics in 37 out of 48 cases based on cost function evaluation, 26 of 48 cases based on feature similarity index, and 20 of 32 cases based on Dice similarity. The obtained results demonstrate that population-based metaheuristics can be successfully applied to entropy-based image thresholding and that strengthening both exploitation and exploration strategies, as performed in ME-GDEAR, is crucial for designing such an algorithm.

## 1. Introduction

Image segmentation is a challenging task in machine vision. It is the process of dividing an image into several non-overlapping areas based on features such as colour or texture. Image segmentation is used in a broad spectrum of applications including medicine [1,2], the modelling of microstructures [3] and food quality [4]. While a variety of image segmentation approaches have been proposed [5] and although deep learning methods have shown impressive performance for image segmentation tasks [6], techniques based on image thresholding remain popular due to their simplicity and robustness [7,8] despite not requiring a training process. Image thresholding aims to find the threshold value(s) for an image using information from the histogram of an image. While bi-level image thresholding (BLIT) methods try to find a single threshold to discriminate between the background and foreground, multi-level image thresholding (MLIT) approaches have determined multiple threshold values to partition an image into several regions. MLIT is a challenging task and has thus attracted the attention of significant research [9,10,11,12].

In recent years, entropy-based MLIT algorithms have been extensively employed for image segmentation [13,14,15]. Entropy is a measure of randomness or disorder so that homogeneous regions are characterised by low unpredictability [16]. A higher value of entropy thus shows higher separability between background and foreground, while different types of entropy, such as Kapur entropy [17], Reni entropy [18], Shannon entropy [19] and Tsallis entropy [20] can be employed. The information considered is either additive or non-additive, and is exploited in entropy-based image thresholding [21]. Renyi entropy can address additivity [18], while Tsallis entropy can take into consideration non-additivity; however, neither can simultaneously employ both additive and non-additive information.

Masi entropy [22] combines the additivity feature of Renyi entropy and the non-extensitivity feature of Tsallis entropy. Masi entropy has shown remarkable performance for BLIT, but its efficiency drastically decreases when increasing the number of thresholds due to the resulting time complexity. To address this issue, population-based metaheuristic algorithms (PBMHs) such as differential evolution (DE) and particle swarm optimisation (PSO), where a population of candidate solutions is iteratively and co-operatively improved, offer a powerful alternative. While PBMHs have been extensively used for image segmentation [23], there are only few works on PBMHs for Masi-based MLIT problems. Khairuzzaman et al. [24] employ PSO with Masi entropy for image segmentation and shows that PSO can outperform the dragonfly algorithm (DA) on six benchmark images. Fractional order Darwinian PSO was used in [25] for image segmentation based on Masi entropy. A post-processing step was introduced to remove small segmented regions and merge them into bigger regions. In [26], the water cycle algorithm (WCA) was employed for image thresholding using Masi entropy as the objective function. The obtained results indicate that WCA can achieve better performance in comparison to 5 other algorithms on 10 benchmark images. Ref. [27] employs the moth swarm algorithm (MSA) for image thresholding based on context-sensitive energy and Masi entropy and shows that it can outperform several PBMHs. Other PBMHs including multi-verse optimiser (MVO) [28,29], Harris hawks optimisation (HHO) [21,30], cuttlefish algorithm (CA) [31], and barnacles mating optimiser (BMO) [32] have also been employed for Masi entropy-based MLIT problems.

Differential evolution [33] is a well-established PBMH with three main operators: mutation, crossover, and selection. Similar to other PBMHs, during initialisation, a starting population of individuals is (randomly) generated. The mutation operator generates a mutant vector based on the differences among individuals, while crossover combines the mutant vector and its parent. Finally, the selection operator chooses the individual to include in the next iteration. In recent years, much research has focussed on improving DE [34,35,36], while DE has been shown to yield notable performance in solving complex problems [37,38,39].

In this paper, we propose a novel multi-level image thresholding algorithm named Masi entropy-based grouping differential evolution boosted by attraction and repulsion strategies (ME-GDEAR). Our proposed algorithm employs a grouping strategy using a clustering algorithm to partition the current population into groups. ME-GDEAR then uses the cluster information to update the current population. In addition, we apply attraction and repulsion strategies to further improve the efficacy of the algorithm. Extensive experiments on a set of benchmark images convincingly show the excellent image thresholding performance of ME-GDEAR in comparison to other approaches.

The remainder of the paper is organised as follows. Section 2 reviews some background about differential evolution, clustering, and image thresholding. Section 3 explains our proposed algorithm in detail, while Section 4 evaluates and discusses the obtained experimental results. Section 5 concludes the paper.

## 2. Background

### 2.1. Differential Evolution

Differential evolution (DE) [33] is a well-established population-based metaheuristic algorithm that has shown good performance in solving complex optimisation problems from a broad spectrum of domains [37,40,41]. The canonical DE algorithm includes four main steps: initialisation, crossover, mutation, and selection. The pseudo-code of DE is given in Algorithm 1, whereas the main operators are described below.

**Algorithm 1:** Pseudo-code of DE algorithm. 
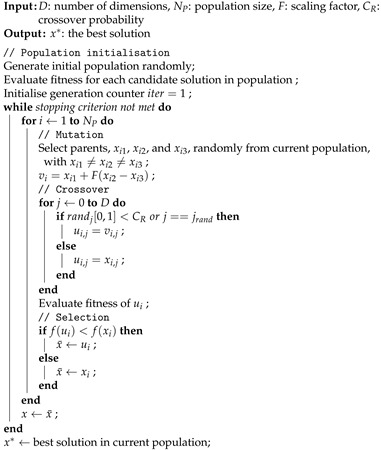


#### 2.1.1. Initialisation

Similarly to other PBMHs, DE begins with a population of $N_{P}$ randomly generated individuals, where for a *D*-dimensional problem, an individual is defined as $x_{i}=\left(x_{i,1},x_{i,2},...,x_{i,D}\right)\in R^{D}$.

#### 2.1.2. Mutation

Mutation creates a mutant vector based on differences among individuals. While there is a wide range of mutation operators, *DE/rand/1* is popular and defined as

(1) $$v_{i}=x_{r1}+F\left(x_{r2}-x_{r3}\right),$$

where $x_{r1}$ (called the base vector), $x_{r2}$, and $x_{r3}$ are three randomly selected individuals distinct from the current population, and *F* is a scaling factor.

#### 2.1.3. Crossover

Crossover combines the mutant and parent vectors, with the aim of enhancing the exploration of the population. Among the different crossover operators, binomial crossover is often chosen and is formulated as

(2) $$u_{i,j}=\left\{\begin{array}{cc} v_{i,j} & rand\left(0,1\right)\leq C_{R}\\ x_{i,j} & \mathrm{otherwise} \end{array}\right.,$$

where $i=1,...,N_{pop}$, j=1,...,D, *u* is called a trial vector, $C_{R}$ is the crossover rate, and $j_{rand}$ is a random number in $\left[1;N_{pop}\right]$.

#### 2.1.4. Selection

The selection operator aims to select the better individual from the trial vector and the parent vector for inclusion in the next population.

### 2.2. Clustering

Clustering is an unsupervised pattern recognition technique to divide a set of samples into a number of groups so that samples located in the same cluster are more similar compared to those in different clusters. The main characteristics of a clustering algorithm are:

- Each cluster should have at least one sample: $C_{i}\neq \phi ,i=1,...,K$;
- The total number of samples in all clusters must be equal to the total number of samples: $\cup_{i=1}^{K}=O$; and
- Distinct clusters should not have a mutual sample: $c_{i}\cap c_{j}=\phi ,j=1,...,K,i\neq j$.

Among the different clustering algorithms, *k*-means [42] is a simple yet effective approach that is widely employed. *k*-means proceeds in the following steps:

1. Randomly select *k* samples as cluster centres;
2. Allocate each sample to its closest cluster centre based on a distance metric (often Euclidean distance);
3. Recalculate the new cluster centres as the mean value of the samples located in each cluster;
4. If the stopping condition is satisfied, the algorithm has terminated—otherwise go to Step 2.

### 2.3. Multi-Level Image Thresholding

Multi-level image thresholding is a popular approach for image segmentation. MLIT aims to find *D* threshold values as

(3) $$\begin{array}{ccc} M_{0} & = & \left\{f\left(x,y\right)\in I|0\leq f\left(x,y\right)\leq th_{1}-1\right\}\\ M_{1} & = & \left\{f\left(x,y\right)\in I|th_{1}\leq f\left(x,y\right)\leq th_{2}-1\right\}\\ M_{i} & = & \left\{f\left(x,y\right)\in I|th_{i}\leq f\left(x,y\right)\leq th_{i+1}-1\right\}\\ M_{D} & = & \left\{f\left(x,y\right)\in I|th_{m}\leq f\left(x,y\right)\leq L-1\right\} \end{array}$$

where f(x,y) indicates an image pixel at location (x,y) and *L* is the number of intensity levels in the image. $M_{i}$ thus gives an image segment based on the threshold values and it is the selection of these $th_{D}$ that is at the core of this paper.

## 3. Proposed ME-GDEAR Algorithm

**Figure 1:**
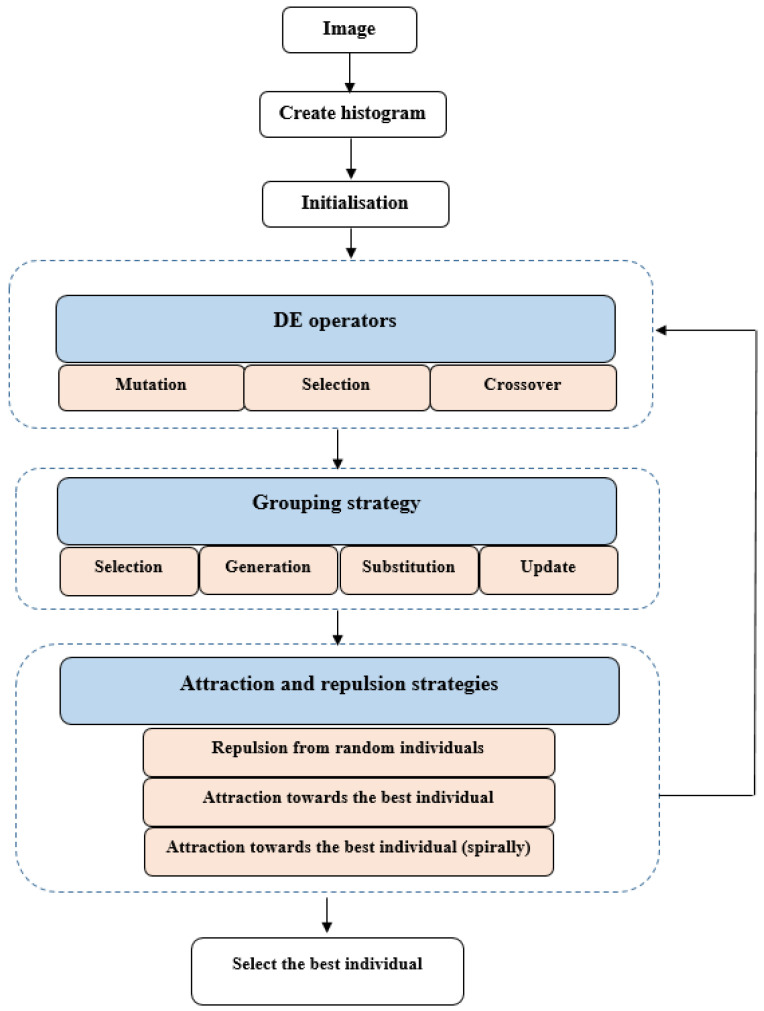
General structure of the ME-GDEAR algorithm.

In this paper, we propose Masi entropy-based grouping differential evolution boosted by attraction and repulsion strategies (ME-GDEAR), as an improved DE algorithm for multi-level image thresholding. The general structure of our proposed algorithm is shown in Figure 1. In the following, we first explain the main components of ME-GDEAR, and then detail how the algorithm proceeds.

### 3.1. Grouping Strategy

We propose a grouping strategy, inspired by [43], for dividing the current population into groups. Our grouping strategy has two main operators: region creation and population update.

#### 3.1.1. Region Creation

**Figure 2:**
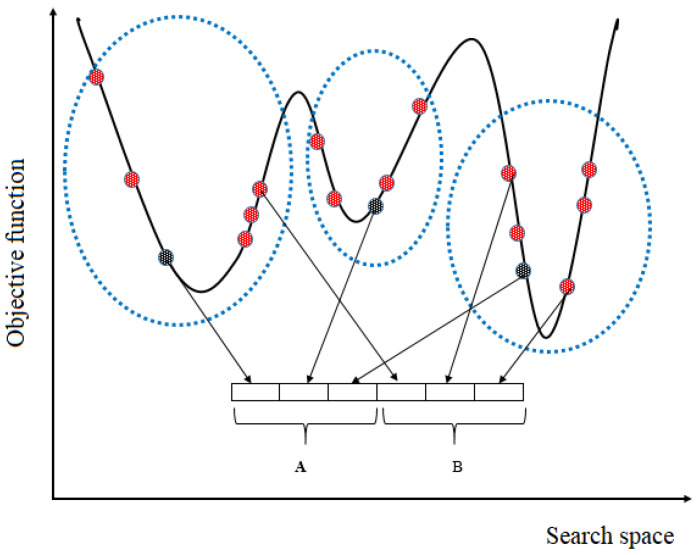
Population clustering: red points represent individuals and black points indicate cluster centres. The population is divided into 3 clusters. A is the set of cluster centres while B contains some random individuals.

Our grouping strategy first creates some regions based on the *k*-means algorithm. Here, each cluster indicates a region and the number of clusters is set as a random number between 2 and $\sqrt{N_{P}}$. Cluster centres are the means of individuals in the same cluster, meaning that each cluster centre holds information about the individuals in the cluster. The cluster centres thus support a sort of multi-parent crossover. Figure 2 indicates the process of region creation for a toy example.

#### 3.1.2. Population Update

The cluster centres created above should be included in the current population. To this end, we employed a generic population-based algorithm (GPBA) proposed in [43,44] to boost the performance of the algorithm. GPBA uses four operators to tackle optimisation problems, namely:

- **Selection:** randomly choose some individuals from the current population. This relates to choosing initial samples in the *k*-means algorithm;
- **Generation**: create *m* individuals as set *A*. For this, ME-GDEAR selects the cluster centres as the new individuals, that is, the new individuals are generated using *k*-means clustering;
- **Substitution**: choose *m* individuals (set *B*) from the population for substitution. There are various ways to select some individuals from the population; ME-GDEAR uses random selection as a simple selection strategy;
- **Update**: from the union set A∪B, the *m* best individuals are selected as $\bar{B}$. The new population is then obtained as $\left(P-B\right)\cup \bar{B}$.

#### 3.1.3. Clustering Period

In ME-GDEAR, clustering is not performed in every iteration. Instead, clustering is periodically performed [43,45], where parameter $C_{P}$ defines the clustering period. Selecting an effective clustering period is essential so that DE can create stable clusters.

### 3.2. Attraction and Repulsion Strategies

We introduce attraction and repulsion strategies into ME-GDEAR inspired by the WOA algorithm [46] in order to explore the search space more effectively. These strategies are applied with a probability $P_{r}$. Three operators are employed, which we explain below, while switching between them is performed based on some probabilities.

#### 3.2.1. Repulsion from Random Individuals

This operator causes all individuals to move away from some randomly selected individuals as

(4) $$x_{i}=x_{r}-AM,$$

with

(5) $$M=|Cx_{r}-x_{i}|,$$

where $x_{r}$ is a random individual selected from the current population, *A* is a number greater than 1, and *C* is a random number between 0 and 2.

#### 3.2.2. Attraction towards the Best Individual

Here, each individual tries to converge towards the best individual as

(6) $$x_{i}=x_{best}-AM,$$

with:

(7) $$M=|Cx_{best}-x_{i}|,$$

where $x_{best}$ is the best individual in the current population, *A* is a number less than 1, and *C* is a random number between 0 and 2.

#### 3.2.3. Attraction towards the Best Individual (Spirally)

This operator updates an individual in a spiral way as

(8) $$x_{i}=x_{best}+e^{bl}\cos\left(2\pi l\right)M,$$

with:

(9) $$M=|x_{best}-x_{i}|,$$

where $x_{best}$ is the position of the best individual, *b* is a constant, and *l* is a random number in [−1,1].

### 3.3. Encoding Strategy

**Figure 3:**
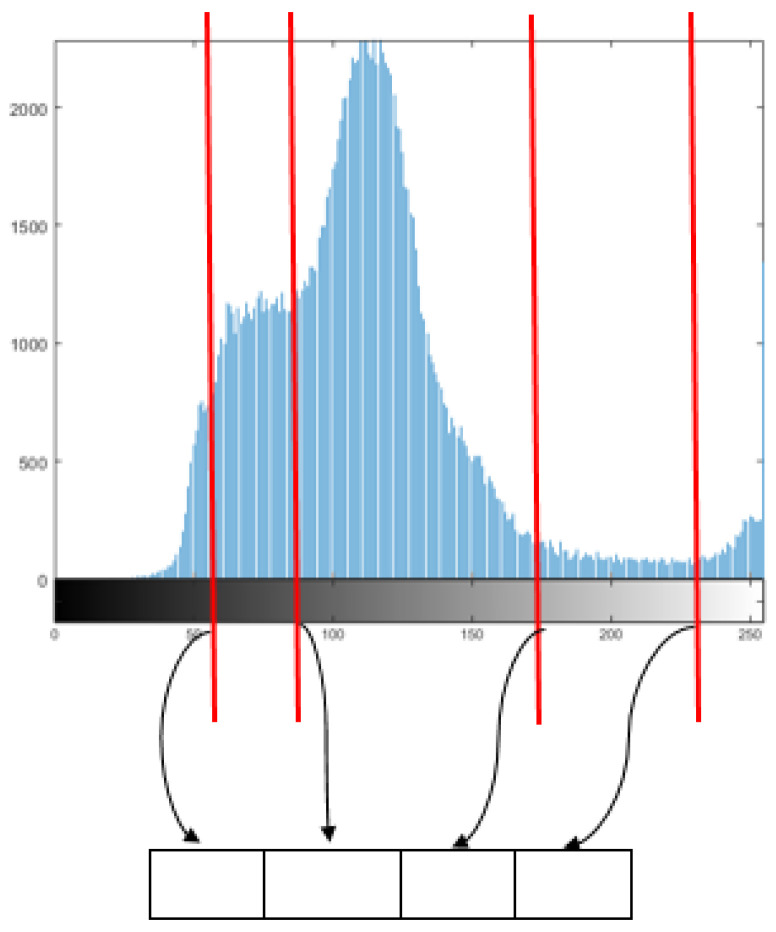
Encoding strategy in ME-GDEAR.

The encoding strategy determines the structure of each individual in the population. In ME-GDEAR, we employed, as illustrated in Figure 3, a one-dimensional vector to encode the threshold values as

(10) $$x=[th_{1},th_{2},...,th_{D}],$$

where *D* is the number of threshold values, and $th_{i}$ is the *i*-th threshold value.

### 3.4. Objective Function

The probability of occurrence of pixel intensity *i* is:

(11) $$h_{i}=\frac{n_{i}}{MN},\;h_{i}\geq 0,\;\sum_{i=0}^{L-1}h_{i}=1,$$

where *M* and *N* are the dimensions of the image, *L* is the number of image intensities, and $n_{i}$ is the number of pixels of intensity *i*.

For our MLIT algorithm, the class likelihoods are computed as

(12) $$w_{1}=\sum_{i=0}^{th_{1}}h_{i},w_{2}=\sum_{i=th_{1}+1}^{th_{2}}h_{i},\cdots ,w_{D}=\sum_{i=th_{D}-1}^{L-1}h_{i},$$

and the multi-level Masi entropy (MME) of each class is calculated as

(13) $$\begin{array}{ccc} H_{1} & = & \frac{1}{1-r}\log\left[1-\left(1-r\right)\sum_{i=0}^{th_{1}}\left(\frac{h_{i}}{w_{1}}\right)\log\left(\frac{h_{i}}{w_{1}}\right)\right]\\ H_{2} & = & \frac{1}{1-r}\log\left[1-\left(1-r\right)\sum_{th_{1}+1}^{th_{2}}\left(\frac{h_{i}}{w_{2}}\right)\log\left(\frac{h_{i}}{w_{2}}\right)\right]\\ \cdots \\ H_{D} & = & \frac{1}{1-r}\log\left[1-\left(1-r\right)\sum_{th_{D}+1}^{L}\left(\frac{h_{i}}{w_{D}}\right)\log\left(\frac{h_{i}}{w_{D}}\right)\right], \end{array}$$

where *r* is the value of the entropic parameter.

Finally, we define the objective function as

(14) $$f\left(t_{1},t_{2},...,t_{D}\right)=H_{1}+H_{2}+...+H_{D}.$$

### 3.5. Proposed Algorithm

Our ME-GDEAR algorithm, which performs clustering-based DE boosted by attraction and repulsion strategies for Masi-entropy multi-level image segmentation, proceeds in the following steps:

1. Initialise the parameters including population size $N_{P}$, maximum number of function evaluations $NFE_{\max}$, clustering period $C_{P}$, probability of attraction and repulsion strategies $P_{r}$, and entropic parameter *r*. Set the current number of function evaluations NFE=0, and the current iteration iter=1.
2. Generate the initial population of size $N_{P}$ using uniformly distributed random numbers.
3. Calculate the objective function value of each individual in the population using Equation (14).
4. Set $NFE=NFE+N_{P}$.
5. For each individual, perform Steps 5a–5d:
  (a) Apply mutation operator;
  (b) Apply crossover operator;
  (c) Calculate the objective function using Equation (14);
  (d) Apply selection operator.
6. Set $NFE=NFE+N_{P}$.
7. If $\left(iter\%C_{P}==0\right)$, go to Step 7a—otherwise, go to Step 8:
  (a) Randomly generate *k* as random integer number between 2 and $\sqrt{N_{P}}$;
  (b) Perform *k*-means clustering and select *k* cluster centres as set *A*;
  (c) Select *k* individuals randomly from current population as set *B*;
  (d) From A∪B, select best *k* individuals as $\bar{B}$;
  (e) Select new population as $\left(P-B\right)\cup \bar{B}$.
8. If $rand<P_{r}$, go to Step 8a—otherwise, go to Step 9.
  (a) Generate two random numbers, r1 and r2, between 0 and 1, and one random number, *C*, between 0 and 2;
  (b) Set *a* as $2-NFE(2/NFE_{max})$ and *A* as 2ar1−a;
  (c) If rand<0.5, go to Step 8d—otherwise, go to Step 8g;
  (d) If |A|≥1, go to Step 8e—otherwise, go to Step 8f;
  (e) Apply repulsion operator using Equation (4) and go to Step 9;
  (f) Apply attraction operator using Equation (6) and go to Step 9;
  (g) Apply spiral attraction operator using Equation (8).
9. Set iter=iter+1.
10. If $NFE>NFE_{\max}$, go to Step 11—otherwise, go to Step 5.
11. Select the best individual as the set of optimal threshold values.

### 3.6. Monte-Carlo Simulations

In our approach, clustering acts similarly to a multi-parent crossover. To analyse the effect of clustering on the algorithm’s performance, we designed some Monte-Carlo simulations. For this, we selected three representative images from the Berkeley image segmentation database [47], namely 147091, 101087, and 253027.

The golden region was defined as a hyper-sphere whose diameter is the middle 60% interval of the shrunken search space and whose centre is the centre of the shrunken search space [48]. The lower and higher bounds of the shrunken search space are the minimum and maximum of the current population, respectively. An individual is located in the golden region if the distance to the centre point is less than the radius of the hyper-sphere. Points in the golden region are more likely to be close to an unknown optimum solution [48].

**Figure 4:**
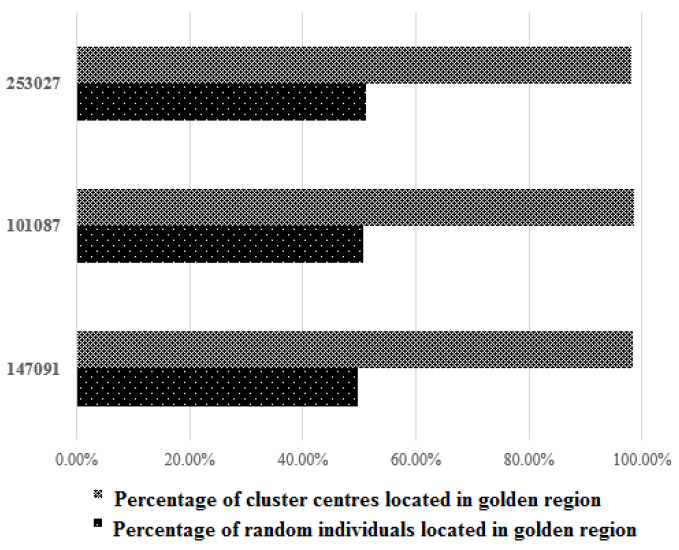
Fractions of cluster centres and random individuals located in the golden region.

In the first simulation, the percentages of cluster centres and random individuals which are located in the golden region were computed. In each iteration, several randomly generated individuals were generated (based on the population size) and their locations were found (inside the golden region or not). Then, the location of cluster centres was obtained. Figure 4 gives the results (all simulations were repeated 10,000,000 times) and shows that the probability of a cluster centre falling in the golden region is much higher than that of a random individual, indicating that cluster centres are biased toward the centre of the golden region.

**Figure 5:**
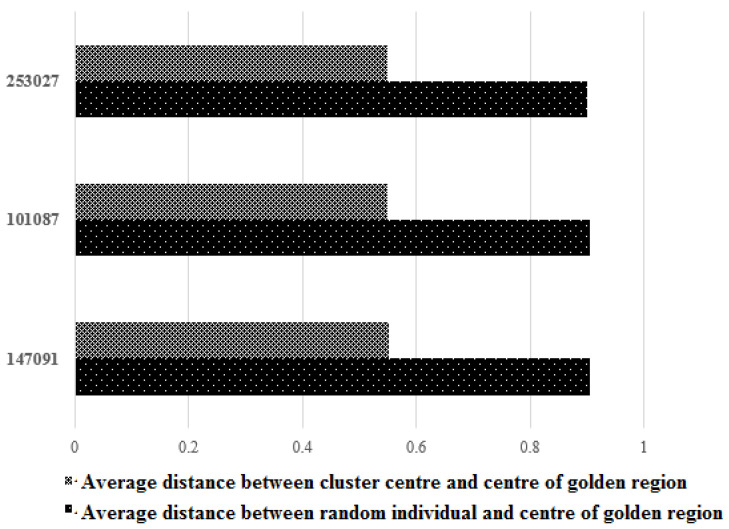
Distance between the centre of the golden region and the cluster centres/random individuals.

In the next experiment, we calculated the distance between the centre of the golden region and cluster centres and between the centre of the golden region and random individuals. From Figure 5, which shows the results, we can observe that the distance between the cluster centres and the centre of golden region is smaller than the distance between random individuals and the centre of golden region, indicating that the cluster centres are closer to the centre of golden region compared to random individuals.

**Figure 6:**
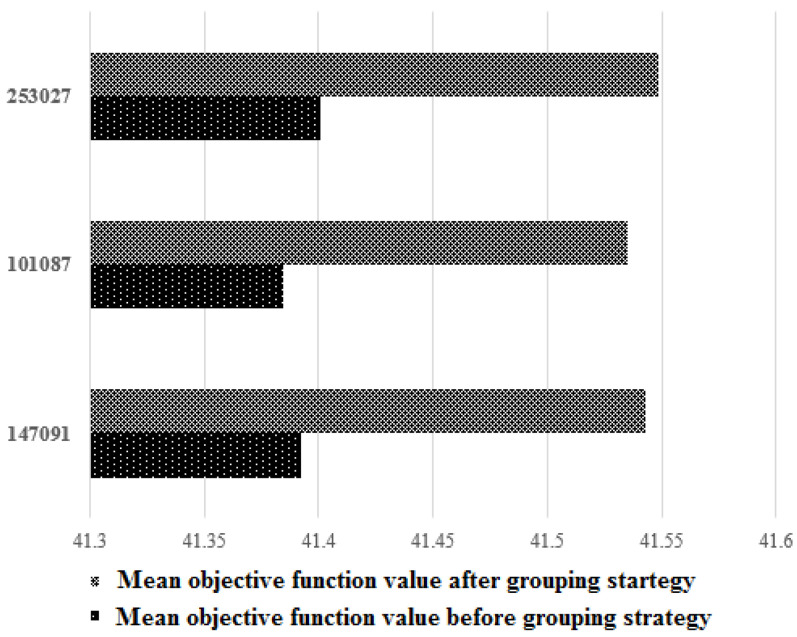
Mean objective function results with/without grouping strategy.

Finally, we evaluate the mean objective function value with and without our proposed grouping strategy to assess its effectiveness. Figure 6 shows that for all images, the mean objective function values are improved, confirming that the grouping stage leads to improved thresholding performance.

## 4. Results and Discussion

**Figure 7:**
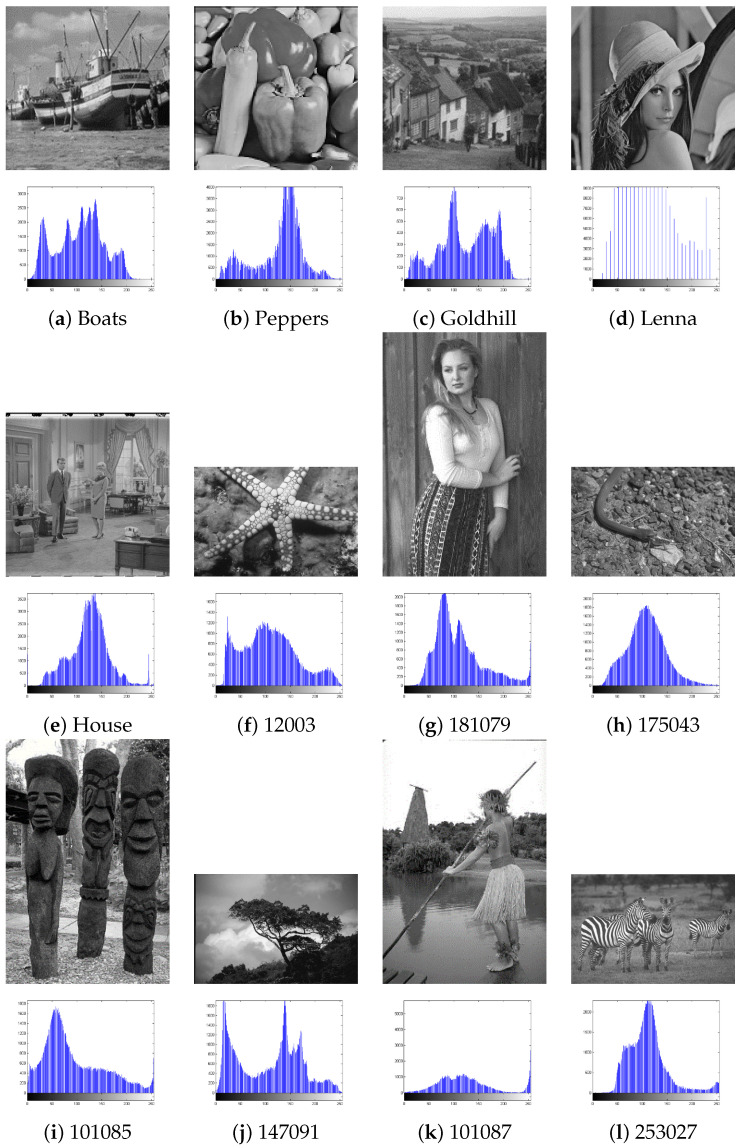
Test images and their histograms.

In order to evaluate the performance of our proposed ME-GDEAR algorithm, we performed several experiments on a set of benchmark images which are widely used to test thresholding algorithms, namely *Boats*, *Peppers*, *Goldhill*, *Lenna*, and *House*, as well as seven images from the Berkeley image segmentation database [47], 12003, 181079, 175043, 101085, 147091, 101087, and 253027. Figure 7 shows the images and their histograms. As we can see, the image histograms show different characteristics; some images such as *Lenna* and *Peppers* have different peaks and valleys, while others such as 175043 have only one peak and images such as *Goldhill* have abrupt changes in the histogram.

We compared ME-GDEAR with a number of population-based image thresholding algorithms, including Masi entropy-based differential evolution (ME-DE), the Masi entropy-based firefly algorithm (ME-FA), Masi entropy-based bat algorithm (ME-BA), Masi entropy-based moth flame optimisation (ME-MFO), Masi entropy-based dragonfly algorithm (ME-DA), and Masi entropy-based whale optimisation algorithm (ME-WOA).

The population size and the number of function evaluations for all algorithms were 50 and 10,000, respectively. For ME-GDEAR, $C_{p}$ and $p_{r}$ are set to 5 and 0.2, respectively. For the other algorithms, we used the default values for the various parameters which are listed in Table 1. For all algorithms, the entropic parameter was set to 1.2. Each algorithm was run 25 times and we reported the average and standard deviation over these 25 runs.

### 4.1. Objective Function Results

We first compared the algorithms in terms of objective function values. Table 2 gives the results of all algorithms and all images for D=3. For each image and algorithm, we give the average, standard deviation, and resulting rank (based on the average) of each algorithm. In addition, the average ranks and overall ranks are reported.

As we can see, ME-GDEAR is ranked first or second for 8 of the 12 images, leading to the first overall rank. ME-DE is ranked top for three images, while ME-FA gives the best results for two images and these two algorithms give the second-best results overall.

Table 3 reports the results for D=4. ME-GDEAR is again clearly ranked first overall. By comparing Table 2 and Table 3, we can observe that ME-DE drops from an average rank of 3.25 to 4.50, leading to an overall rank of 5 for D=4. In contrast, ME-MFO is ranked second overall for D=4, improving from its fourth rank for D=3.

For D=5, similar results can be seen in Table 4. ME-GDEAR yields the first overall rank, while ME-MFO is ranked second. There is a clear difference between the average rank of ME-GDEAR (1.83) and that of ME-DE (4.17) which shows that our approach clearly outperforms differential evolution.

The curse of dimensionality is a challenging problem in solving an optimisation problem, since increasing the number of dimensions results in exponentially expanding the search space. To assess our proposed algorithm in higher dimensions, we compared ME-GDEAR for D=10 against the other algorithms in Table 5. It is obvious that our algorithm again yields the best results, being ranked first or second for 9 of the 12 images, while ME-BA is ranked second overall.

Overall, ME-GDEAR thus outperforms all other algorithms for all tested dimensionalities, indicating the impressive multi-level image thresholding performance.

### 4.2. Feature Similarity Index Results

The feature similarity index measure (FSIM) [53] is a popular measure for evaluating image quality which is based on two low-level features—phase congruency, which measures the significance of local structures; and gradient magnitude, which incorporates contrast information.

Table 6 lists the FSIM results for D=3. From there, we can see that our proposed algorithm is again ranked top overall. The same holds for D=4 whose results are in Table 7 and for D=5 with results in Table 8.

The results for the higher-dimensional problem with D=10 are given in Table 9. From there, we can see that ME-GDEAR maintains its efficacy and outperforms all other algorithms.

Overall, ME-GDEAR also outperforms all other algorithms in terms of FSIM and does so for all dimensionalities, confirming the efficacy of our proposed algorithm.

### 4.3. Dice Measure

We further performed an evaluation based on Dice similarity [54], which measures the overlap between two segmented images. Since the Dice measure requires a ground truth, we can only apply it on the images of the Berkeley segmentation dataset. As there are multiple manual segmentations for each image, we take the maximum obtained Dice score as our measure for comparison.

Table 10 gives the results for D=3 and shows ME-GDEAR to give the best Dice score for 5 of the 7 images, and, consequently, the best average rank.

Similar results are obtained for D=4, D=5, and D=10, as can be observed from Table 11, Table 12 and Table 13, respectively.

### 4.4. Statistical Tests

Owing to the random characteristics of PBMHs, we also performed statistical tests, based on objective function performance, to further assess the algorithms. In particular, we conducted two non-parametric statistical tests, the Wilcoxon signed rank test and the Friedman test [55]. The Wilcoxon signed rank test is a pair-wise test to compare two algorithms, while the Friedman test allows to evaluate more than two algorithms. The null hypothesis ($H_{0}$) states that there is no significant difference between algorithms, while the alternative hypothesis ($H_{1}$) investigates a difference. Furthermore, the level of statistical significance α indicates the hypothesis rejection probability: if the calculated *p*-value is lower than α, $H_{0}$ is rejected.

The results of the Wilcoxon signed rank test between ME-GDEAR and the other algorithms are given in Table 14. From there, we can see that in all cases, the obtained *p*-value is much smaller than α=0.05, confirming that ME-GDEAR statistically outperforms the other algorithms.

The results of the Friedman test are given in Table 15. It is apparent that ME-GDEAR yields the lowest rank (1.96) and with a wide margin over the second ranked algorithm (ME-BA). The obtained *p*-value is negligible, confirming the fact that there is a significant difference between the algorithms. The critical value for (8 − 1) = 7 degrees of freedom with a 0.05 significance level is 14.067 (from chi-squared distribution table). The obtained chi-squared value of 87.6 is much higher than the critical value; in other words, $H_{0}$ is rejected.

### 4.5. Visual Evaluation

In this section, we visually compare the results of the algorithms. For this, we select (due to length restrictions) image 147091 for D=5 and image 101087 for D=10 as representatives examples. Since the images are from the Berkley segmentation dataset, there are several ground truth segmentations available for each, although these are often quite different.

**Figure 8:**
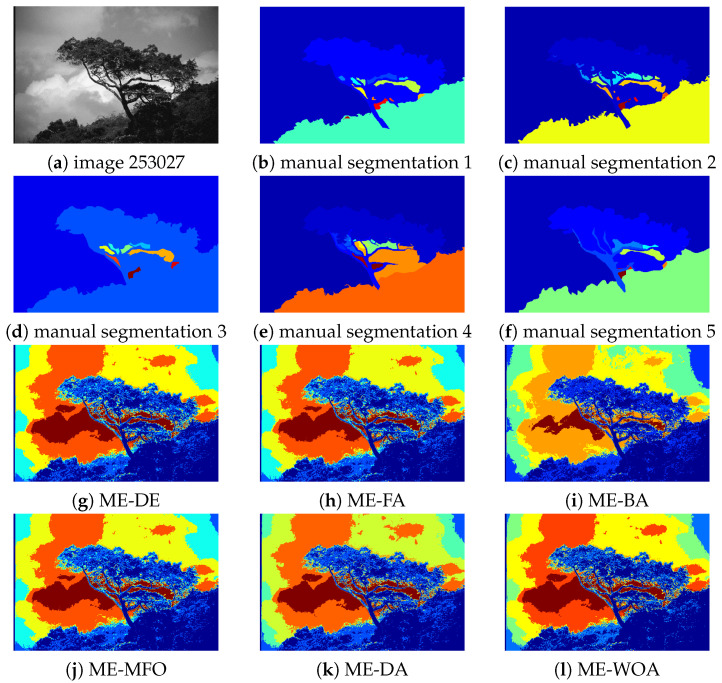

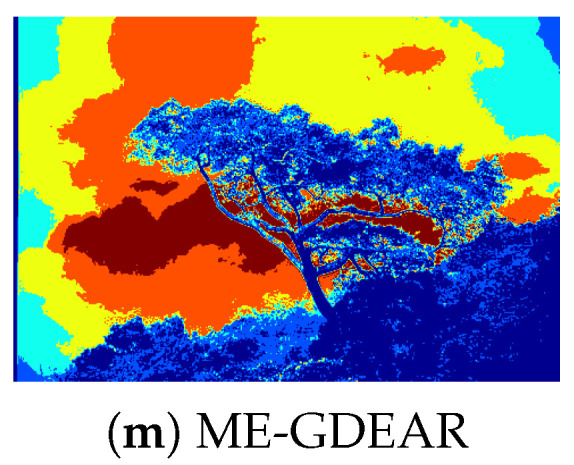
Thresholding results for image 147091 for D=5. (**a**) Original image, (**b**–**f**) true manual segmentation, (**g**) segmented image for ME-DE, (**h**) segmented image for ME-FA, (**i**) segmented image for ME-BA, (**j**) segmented image for ME-MFO, (**k**) segmented image ME-DA, (**l**) segmented image for ME-WOA, and (**m**) segmented image for ME-GDEAR.

Figure 8 shows the manual segmentations together with the images thresholded by all algorithms for image 147091 for D=5. We can notice that our proposed algorithm can segment the image with less noise, particularly the parts of the sky that are cloudless. In contrast, some algorithms such as ME-BA and ME-WOA are unable to distinguish between the left vertical margin and its adjacent parts.

**Figure 9:**
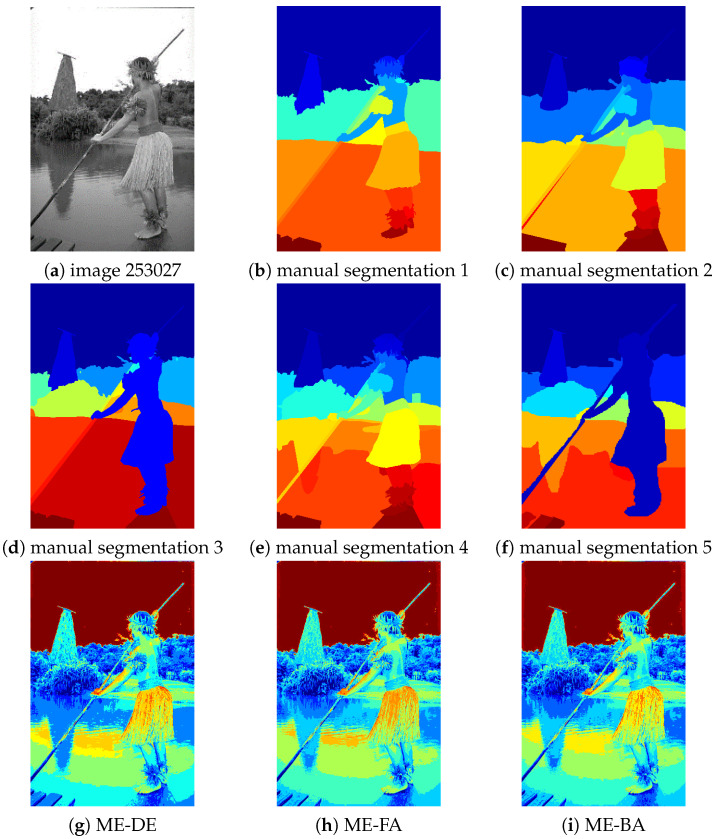

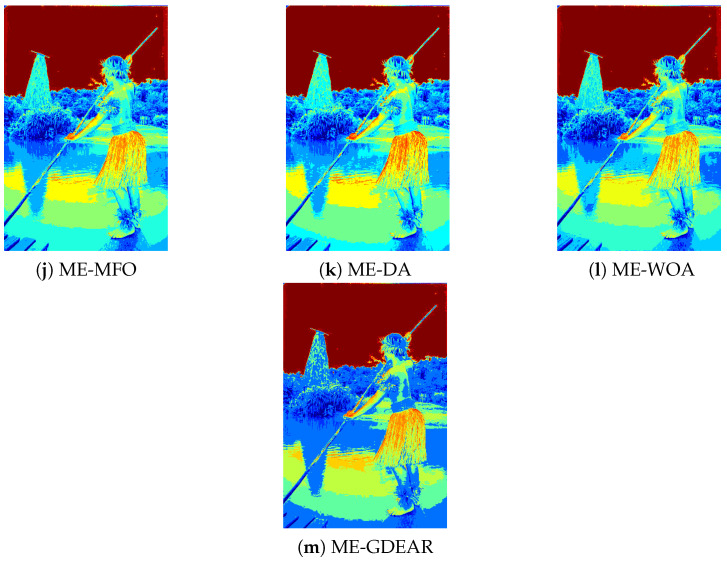
Thresholding results for image 101087 for D=10. (**a**): original image, (**b**–**f**): different manual segmentations, (**g**) segmented image for ME-DE, (**h**) segmented image for ME-FA,(**i**) segmented image for ME-BA, (**j**) segmented image for ME-MFO, (**k**) segmented image for ME-DA, (**l**) segmented image for ME-WOA, and (**m**) segmented image for ME-GDEAR.

Figure 9 shows the results for images 101087 and D=10. Here, we can observe that some algorithms such as ME-WOA and ME-BA do not perform well, most noticeably in the sky part, while ME-GDEAR works significantly better and with less noise. Some algorithms such as ME-FA, ME-BA, and ME-WOA cannot properly segment the shadow part of the lake; these algorithms segment the shadow part into three different regions with almost the same proportions, while our proposed algorithm segments this part more reasonably into two partitions. It is worth noting that in our proposed method the distribution of the classes in the shadow part is not the same and most of the shadow part belongs to one single class, which is more in line with reality.

### 4.6. Effect of Parameters

**Figure 10:**
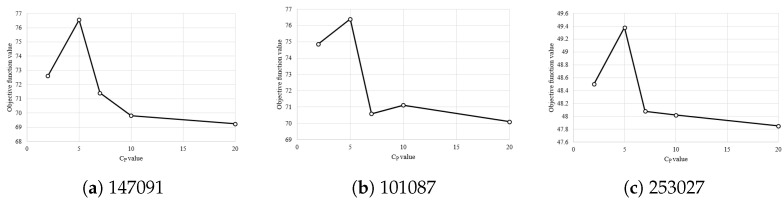
Effect of $C_{P}$ on the mean objective function value for images (**a**) 147091, (**b**) 101087, and (**c**) 253027 for D=10.

In ME-GDEAR, we introduce two new parameters, namely $C_{P}$ and $P_{r}$. To see their effect, we select three representative images, 147091, 101087, and 253027 with D=10. As shown in Figure 10, the performance highly depends on $C_{P}$. Therefore, finding a good value for $C_{P}$ is beneficial to achieve better thresholding. The best value was obtained for $C_{P}=5$.

**Figure 11:**
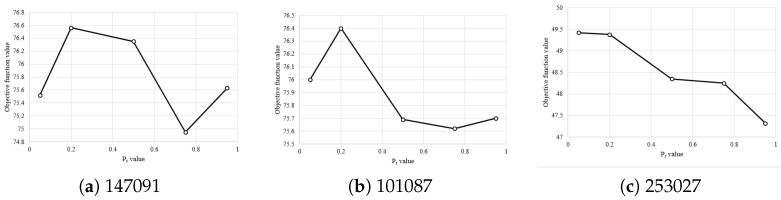
Effect of $P_{r}$ on the mean objective function value for images (**a**) 147091, (**b**) 101087, and (**c**) 253027 for D=10.

Figure 11 shows results for different values of $P_{r}$. As we can see, 0.2 is an appropriate value for this parameter.

## 5. Conclusions

Multi-level image thresholding remains a popular image segmentation approach. Its aim is to find optimal thresholds based on information available in the image histogram. In this paper, we proposed an improved differential evolution algorithm for MLIT based on Masi entropy. Our ME-GDEAR algorithm introduces (1) a grouping strategy into DE to cluster the population and use cluster information to update the population; and (2) attraction and repulsion strategies to more effectively update individuals. Experiments on a benchmark image set with different characteristics clearly demonstrate that ME-GDEAR outperforms other MLIT approaches.

One challenge of image thresholding algorithm is that they may not be too widely used on their own, particularly for higher dimensions. However, they can also be effectively employed as a pre-processing technique. For example, ref. [56] uses image thresholding as a pre-processing step for the application of a subsequent graph cut segmentation algorithm. Therefore, in future work, we intend to integrate our approach with other image segmentation algorithms. Another challenge is that only the image histogram is used, thus ignoring 2-dimensional image information including texture.

Furthermore, some of the drawbacks of ME-GDEAR can be addressed in future work. For instance, it uses *k*-means to cluster the population which can be time-consuming. Using methods with lower computational demand can thus be considered. Furthermore, as is common with other population-based metaheuristic algorithms, parameter tuning is a demanding task and investigating mechanisms for automatic parameter-tuning will be beneficial. Other planned future work includes the application of alternative objective functions to improve segmentation and a multi-objective variant of the algorithm.

## Figures and Tables

**Table 1:** Parameter settings for the experiments.

Algorithm	Parameter	Value
ME-DE [33]	scaling factor	0.5
	crossover probability	0.9
ME-FA [49]	light absorption coefficient (γ)	1
	attractiveness at r=0 ($\beta_{0}$)	1
	scaling factor	0.25
ME-BA [50]	loudness	0.5
	pulse rate	0.5
ME-MFO [51]	*a*	−1
	*b*	1
ME-DA [52]	no parameters	
ME-WOA [46]	constant defining shape of logarithmic spiral	1
ME-GDEAR	scaling factor	0.5
	crossover probability	0.9
	clustering period	0.5
	$P_{r}$	0.2

**Table 2:** Objective function results for D=3.

Image		ME-DE	ME-FA	ME-BA	ME-MFO	ME-DA	ME-WOA	ME-GDEAR
Boats	mean	35.23	34.25	33.98	34.85	34.73	34.45	34.71
	std.dev.	0.02	0.97	0.96	0.49	0.54	0.41	0.64
	rank	1	6	7	2	3	5	4
Peppers	mean	66.20	63.92	58.27	64.23	66.42	61.71	66.33
	std.dev.	8.75	9.26	7.95	6.94	7.98	10.34	6.51
	rank	3	5	7	4	1	6	2
Goldhill	mean	15.56	15.77	15.28	16.03	15.76	15.42	16.05
	std.dev.	0.21	0.57	0.93	0.12	0.31	0.89	0.03
	rank	5	3	7	2	4	6	1
Lenna	mean	70.74	64.87	62.04	65.91	61.05	63.54	67.10
	std.dev.	2.17	5.39	5.83	5.67	4.92	6.56	5.04
	rank	1	4	6	3	7	5	2
House	mean	64.75	66.38	64.67	64.43	64.69	65.16	66.64
	std.dev.	2.92	7.13	3.44	1.5cm7	4.10	7.84	4.36
	rank	4	2	6	7	5	3	1
12003	mean	66.30	62.38	58.57	62.06	64.88	64.44	64.29
	std.dev.	6.61	6.47	5.77	7.10	5.17	6.29	7.17
	rank	1	5	7	6	2	3	4
181079	mean	66.24	63.42	60.68	67.24	61.74	61.07	63.29
	std.dev.	3.44	7.56	5.97	3.94	5.25	7.62	6.61
	rank	2	3	7	1	5	6	4
175043	mean	63.16	65.59	59.16	63.62	62.32	61.72	64.77
	std.dev.	3.50	6.04	4.16	4.75	5.49	6.50	6.30
	rank	4	1	7	3	5	6	2
101085	mean	63.96	62.49	61.59	64.08	66.85	61.21	66.20
	std.dev.	4.86	5.71	5.09	5.41	3.05	5.94	5.69
	rank	4	5	6	3	1	7	2
147091	mean	67.88	67.97	65.16	67.62	66.95	65.15	68.05
	std.dev.	1.56	2.70	3.82	1.61	1.20	4.40	2.22
	rank	3	2	6	4	5	7	1
101087	mean	59.46	65.73	60.56	64.92	63.64	64.99	71.12
	std.dev.	7.20	9.02	7.60	7.09	7.42	8.55	3.91
	rank	7	2	6	4	5	3	1
253027	mean	29.99	30.07	29.87	30.03	29.92	29.97	30.03
	std.dev.	0.07	0.07	0.23	0.13	0.17	0.16	0.13
	rank	4	1	7	2	6	5	3
average rank		3.25	3.25	6.58	3.42	4.08	5.17	2.25
overall rank		2.5	2.5	7	4	5	6	1

**Table 3:** Objective function results for D=4.

Image		ME-DE	ME-FA	ME-BA	ME-MFO	ME-DA	ME-WOA	ME-GDEAR
Boats	mean	35.39	35.84	35.22	35.87	35.79	35.80	35.84
	std.dev.	0.20	0.02	0.48	0.11	0.19	0.21	0.02
	rank	6	3	7	1	5	4	2
Peppers	mean	66.45	65.67	62.46	68.23	66.39	64.90	71.57
	std.dev.	6.49	10.13	8.86	7.65	7.90	9.98	7.94
	rank	3	5	7	2	4	6	1
Goldhill	mean	16.80	17.32	17.24	18.14	17.21	16.87	17.61
	std.dev.	0.47	0.29	0.56	0.42	0.31	0.75	0.35
	rank	7	3	4	1	5	6	2
Lenna	mean	71.57	66.49	63.34	68.87	63.74	65.17	70.22
	std.dev.	2.06	5.90	5.44	5.58	5.09	6.61	5.22
	rank	1	4	7	3	6	5	2
House	mean	64.92	67.11	64.48	67.14	65.55	64.82	68.62
	std.dev.	2.41	8.91	2.61	4.27	3.78	7.53	4.19
	rank	5	3	7	2	4	6	1
12003	mean	65.84	63.35	63.79	66.29	69.58	64.16	68.69
	std.dev.	7.14	7.49	6.69	6.40	3.66	7.12	5.71
	rank	4	7	6	3	1	5	2
181079	mean	68.29	68.97	60.33	68.33	65.18	62.18	65.78
	std.dev.	2.79	5.29	5.14	4.08	5.40	7.61	6.79
	rank	3	1	7	2	5	6	4
175043	mean	62.84	67.97	61.56	63.57	62.61	59.77	66.16
	std.dev.	3.63	5.65	4.35	3.52	5.77	6.05	6.07
	rank	4	1	6	3	5	7	2
101085	mean	64.24	64.36	62.44	66.87	69.09	65.96	68.04
	std.dev.	4.22	5.80	4.35	5.69	1.54	5.86	4.98
	rank	6	5	7	3	1	4	2
147091	mean	70.11	70.11	68.22	69.73	69.41	66.69	70.68
	std.dev.	2.28	3.40	4.16	1.88	1.64	5.46	2.28
	rank	2	3	6	4	5	7	1
101087	mean	62.25	69.13	62.67	68.57	69.01	66.49	70.57
	std.dev.	5.94	10.06	6.96	7.21	8.01	9.04	7.82
	rank	7	2	6	4	3	5	1
253027	mean	32.99	33.22	32.89	33.26	33.18	33.06	33.21
	std.dev.	0.09	0.11	0.27	0.02	0.11	0.21	0.15
	rank	6	2	7	1	4	5	3
average rank		4.50	3.25	6.42	2.42	4.00	5.50	1.92
overall rank		5	3	7	2	4	6	1

**Table 4:** Objective function results for D=5.

Image		ME-DE	ME-FA	ME-BA	ME-MFO	ME-DA	ME-WOA	ME-GDEAR
Boats	mean	37.76	38.39	37.94	38.40	38.18	38.15	38.35
	std.dev.	0.22	0.12	0.36	0.18	0.20	0.27	0.20
	rank	7	2	6	1	4	5	3
Peppers	mean	68.26	68.96	65.50	69.32	64.66	66.02	70.44
	std.dev.	7.76	7.89	8.12	6.85	7.07	10.10	8.67
	rank	4	3	6	2	7	5	1
Goldhill	mean	17.48	18.82	18.28	19.86	18.61	18.49	19.01
	std.dev.	0.64	0.64	0.71	0.30	0.41	0.46	0.28
	rank	7	3	6	1	4	5	2
Lenna	mean	73.16	68.20	68.15	70.58	64.39	67.49	71.51
	std.dev.	1.51	5.73	5.63	5.42	5.67	7.35	5.43
	rank	1	4	5	3	7	6	2
House	mean	67.70	42.00	64.87	65.34	61.41	58.10	68.78
	std.dev.	3.36	12.44	3.87	9.24	7.85	8.57	4.30
	rank	2	7	4	3	5	6	1
12003	mean	68.55	69.43	65.12	68.96	67.90	64.66	71.27
	std.dev.	4.65	7.55	8.30	7.07	4.65	6.83	5.62
	rank	4	2	6	3	5	7	1
181079	mean	70.16	53.32	62.10	69.29	63.59	61.53	66.81
	std.dev.	3.02	16.02	5.33	8.51	5.27	9.09	5.89
	rank	1	7	6	2	4	5	3
175043	mean	63.96	54.01	61.11	64.55	59.43	60.84	68.18
	std.dev.	4.80	13.72	3.48	4.80	4.70	6.85	6.20
	rank	3	7	4	2	6	5	1
101085	mean	67.46	65.38	67.37	69.57	69.85	66.43	69.95
	std.dev.	4.39	5.28	5.68	4.71	3.45	6.23	4.67
	rank	4	7	5	3	2	6	1
147091	mean	70.68	71.75	67.48	70.72	70.01	69.16	70.73
	std.dev.	1.67	4.51	3.65	4.44	2.49	5.00	1.49
	rank	4	1	7	3	5	6	2
101087	mean	65.94	72.41	64.94	71.27	67.22	68.94	74.58
	std.dev.	7.50	8.27	5.43	8.13	7.40	9.07	5.51
	rank	6	2	7	3	5	4	1
253027	mean	35.87	36.29	36.02	36.28	36.16	36.25	36.22
	std.dev.	0.15	0.05	0.34	0.10	0.16	0.17	0.17
	rank	7	1	6	2	5	3	4
average rank		4.17	3.83	5.67	2.33	4.92	5.25	1.83
overall rank		4	3	7	2	5	6	1

**Table 5:** Objective function results for D=10.

Image		ME-DE	ME-FA	ME-BA	ME-MFO	ME-DA	ME-WOA	ME-GDEAR
Boats	mean	50.61	51.20	51.02	51.38	50.54	51.14	51.10
	std.dev.	0.16	0.15	0.22	0.11	0.27	0.16	0.24
	rank	6	2	5	1	7	3	4
Peppers	mean	51.27	49.84	60.95	54.62	50.92	58.53	73.47
	std.dev.	3.30	0.26	9.31	10.81	6.36	6.73	5.87
	rank	5	7	2	4	6	3	1
Goldhill	mean	24.21	24.45	24.22	26.85	23.16	23.72	24.13
	std.dev.	0.45	1.12	1.55	1.06	0.55	0.87	0.92
	rank	4	2	3	1	7	6	5
Lenna	mean	58.84	49.93	70.03	54.82	50.07	60.99	74.63
	std.dev.	7.21	0.23	5.47	10.61	2.08	5.09	6.31
	rank	4	7	2	5	6	3	1
House	mean	49.51	50.06	63.28	50.29	49.22	51.92	64.51
	std.dev.	0.21	0.17	6.97	0.05	0.32	5.59	8.08
	rank	6	5	2	4	7	3	1
12003	mean	61.17	51.75	68.35	63.73	52.08	60.81	74.15
	std.dev.	5.80	0.12	8.73	12.25	1.82	3.68	6.18
	rank	4	7	2	3	6	5	1
181079	mean	50.30	50.74	64.51	51.17	49.90	58.92	63.68
	std.dev.	0.36	0.39	6.95	0.03	0.52	4.47	5.65
	rank	6	5	1	4	7	3	2
175043	mean	50.84	51.12	61.65	51.72	50.49	56.54	62.62
	std.dev.	0.20	0.32	4.57	0.15	0.41	4.30	6.13
	rank	6	5	2	4	7	3	1
101085	mean	60.09	52.61	68.84	58.63	55.00	66.96	74.33
	std.dev.	7.01	0.14	7.57	8.43	5.81	4.63	4.24
	rank	4	7	2	5	6	3	1
147091	mean	56.56	52.43	69.47	53.44	52.45	67.77	76.56
	std.dev.	6.64	0.13	5.98	4.03	2.52	4.69	3.45
	rank	4	7	2	5	6	3	1
101087	mean	55.69	50.39	62.74	56.56	49.60	56.76	76.40
	std.dev.	6.80	0.14	9.65	11.74	0.33	11.03	8.46
	rank	5	6	2	4	7	3	1
253027	mean	48.80	49.35	49.37	49.51	48.40	49.39	49.38
	std.dev.	0.22	0.16	0.23	0.06	0.35	0.11	0.12
	rank	6	5	4	1	7	2	3
average rank		5.00	5.42	2.42	3.42	6.58	3.33	1.83
overall rank		5	6	2	4	7	3	1

**Table 6:** FSIM results for D=3.

Image		ME-DE	ME-FA	ME-BA	ME-MFO	ME-DA	ME-WOA	ME-GDEAR
Boats	mean	0.4784	0.5276	0.5365	0.4737	0.4713	0.4662	0.4855
	std.dev.	0.0006	0.1130	0.1262	0.0083	0.0085	0.0077	0.0554
	rank	4	2	1	5	6	7	3
Peppers	mean	0.6064	0.5988	0.6048	0.6034	0.5947	0.6089	0.6120
	std.dev.	0.0196	0.0179	0.0175	0.0184	0.0175	0.0189	0.0164
	rank	3	6	4	5	7	2	1
Goldhill	mean	0.6152	0.6237	0.6326	0.5951	0.6206	0.6089	0.6258
	std.dev.	0.0565	0.0513	0.0418	0.0521	0.0562	0.0352	0.0036
	rank	5	3	1	7	4	6	2
Lenna	mean	0.6381	0.6203	0.6129	0.6092	0.6112	0.6219	0.6237
	std.dev.	0.0109	0.0271	0.0271	0.0262	0.0271	0.0263	0.0260
	rank	1	4	5	7	6	3	2
House	mean	0.4519	0.4575	0.4512	0.4484	0.4524	0.4563	0.4537
	std.dev.	0.0137	0.0141	0.0118	0.0105	0.0133	0.0146	0.0138
	rank	5	1	6	7	4	2	3
12003	mean	0.5288	0.5267	0.5343	0.5329	0.5118	0.5182	0.5327
	std.dev.	0.0214	0.0273	0.0276	0.0232	0.0206	0.0239	0.0309
	rank	4	5	1	2	7	6	3
181079	mean	0.5123	0.5169	0.5152	0.5140	0.5120	0.5141	0.5138
	std.dev.	0.0029	0.0048	0.0050	0.0028	0.0016	0.0039	0.0028
	rank	6	1	2	4	7	3	5
175043	mean	0.2920	0.2918	0.2911	0.2917	0.2918	0.2923	0.2948
	std.dev.	0.0033	0.0020	0.0045	0.0033	0.0028	0.0027	0.0023
	rank	3	4	7	6	5	2	1
101085	mean	0.5475	0.5748	0.5862	0.5853	0.5607	0.5590	0.5631
	std.dev.	0.0294	0.0462	0.0485	0.0477	0.0380	0.0445	0.0434
	rank	7	3	1	2	5	6	4
147091	mean	0.5974	0.6270	0.6138	0.6018	0.5940	0.6541	0.6022
	std.dev.	0.0126	0.0591	0.0546	0.0341	0.0016	0.0795	0.0207
	rank	6	2	3	5	7	1	4
101087	mean	0.6353	0.6323	0.6282	0.6338	0.6349	0.6297	0.6384
	std.dev.	0.0076	0.0134	0.0146	0.0111	0.0098	0.0156	0.0025
	rank	2	5	7	4	3	6	1
253027	mean	0.6052	0.6169	0.6348	0.6173	0.6137	0.6154	0.6171
	std.dev.	0.0113	0.0007	0.0462	0.0012	0.0060	0.0062	0.0015
	rank	7	4	1	2	6	5	3
average rank		4.41	3.33	3.25	4.58	5.50	4.08	2.66
overall rank		5	3	2	6	7	4	1

**Table 7:** FSIM results for D=4.

Image		ME-DE	ME-FA	ME-BA	ME-MFO	ME-DA	ME-WOA	ME-GDEAR
Boats	mean	0.7608	0.7674	0.7993	0.7549	0.7362	0.7465	0.7661
	std.dev.	0.0870	0.0031	0.0272	0.0580	0.0982	0.0836	0.0023
	rank	4	2	1	5	7	6	3
Peppers	mean	0.6094	0.6099	0.6057	0.6040	0.5991	0.6016	0.6098
	std.dev.	0.0161	0.0201	0.0197	0.0202	0.0151	0.0205	0.0212
	rank	3	1	4	5	7	6	2
Goldhill	mean	0.6856	0.6961	0.6928	0.6126	0.6783	0.6698	0.6937
	std.dev.	0.0779	0.0745	0.0526	0.0488	0.0808	0.0787	0.0803
	rank	4	1	3	7	5	6	2
Lenna	mean	0.6329	0.6080	0.6028	0.6141	0.6199	0.6207	0.6288
	std.dev.	0.0193	0.0267	0.0237	0.0266	0.0270	0.0266	0.0234
	rank	1	6	7	5	4	3	2
House	mean	0.4461	0.4617	0.4487	0.4564	0.4518	0.4531	0.4573
	std.dev.	0.0076	0.0166	0.0105	0.0148	0.0136	0.0140	0.0146
	rank	7	1	6	3	5	4	2
12003	mean	0.5347	0.5500	0.5372	0.5353	0.5089	0.5391	0.5324
	std.dev.	0.0221	0.0208	0.0267	0.0247	0.0194	0.0268	0.0236
	rank	5	1	3	4	7	2	6
181079	mean	0.5124	0.5153	0.5163	0.5148	0.5142	0.5160	0.5178
	std.dev.	0.0022	0.0040	0.0061	0.0044	0.0034	0.0044	0.0023
	rank	7	4	2	5	6	3	1
175043	mean	0.2925	0.2904	0.2924	0.2926	0.2913	0.2924	0.2924
	std.dev.	0.0028	0.0033	0.0034	0.0028	0.0034	0.0034	0.0028
	rank	2	7	4	1	6	5	3
101085	mean	0.5573	0.5858	0.6029	0.6112	0.5750	0.5793	0.5761
	std.dev.	0.0354	0.0574	0.0577	0.0511	0.0397	0.0474	0.0457
	rank	7	3	2	1	6	4	5
147091	mean	0.6045	0.6406	0.6226	0.6095	0.6034	0.6438	0.6204
	std.dev.	0.0210	0.0570	0.0540	0.0398	0.0197	0.0701	0.0449
	rank	6	2	3	5	7	1	4
101087	mean	0.6398	0.6292	0.6334	0.6383	0.6392	0.6289	0.6366
	std.dev.	0.0082	0.0171	0.0116	0.0094	0.0077	0.0162	0.0116
	rank	1	6	5	3	2	7	4
253027	mean	0.6512	0.6439	0.7278	0.6341	0.6456	0.7124	0.6538
	std.dev.	0.0233	0.0366	0.0807	0.0070	0.0371	0.0856	0.0592
	rank	4	6	1	7	5	2	3
average rank		4.25	3.33	3.41	4.25	5.58	4.08	3.08
overall rank		5.5	2	3	5.5	7	4	1

**Table 8:** FSIM results for D=5.

Image		ME-DE	ME-FA	ME-BA	ME-MFO	ME-DA	ME-WOA	ME-GDEAR
Boats	mean	0.8391	0.8105	0.8440	0.8158	0.8282	0.8374	0.8440
	std.dev.	0.0302	0.0188	0.0407	0.0272	0.0279	0.0368	0.0278
	rank	3	7	1	6	5	4	2
Peppers	mean	0.6098	0.6067	0.6107	0.6141	0.6020	0.6010	0.6140
	std.dev.	0.0169	0.0183	0.0157	0.0131	0.0173	0.0201	0.0208
	rank	4	5	3	1	6	7	2
Goldhill	mean	0.7417	0.7613	0.7859	0.6458	0.7097	0.7432	0.7860
	std.dev.	0.0879	0.0581	0.0625	0.0770	0.0768	0.0761	0.0505
	rank	5	3	2	7	6	4	1
Lenna	mean	0.6400	0.6028	0.6086	0.6084	0.6082	0.6249	0.6358
	std.dev.	0.0105	0.0242	0.0251	0.0259	0.0267	0.0258	0.0187
	rank	1	7	4	5	6	3	2
House	mean	0.4565	0.4524	0.4524	0.4877	0.4678	0.4465	0.4565
	std.dev.	0.0149	0.1245	0.0108	0.1083	0.0807	0.0083	0.0149
	rank	4	6	5	1	2	7	3
12003	mean	0.5221	0.5483	0.5273	0.5494	0.5140	0.5422	0.5428
	std.dev.	0.0200	0.0219	0.0302	0.0208	0.0192	0.0257	0.0296
	rank	6	2	5	1	7	4	3
181079	mean	0.5129	0.6074	0.5151	0.5242	0.5141	0.5175	0.5260
	std.dev.	0.0021	0.1071	0.0051	0.0456	0.0042	0.0049	0.0050
	rank	7	1	5	3	6	4	2
175043	mean	0.2919	0.2917	0.2934	0.2922	0.2916	0.2908	0.2911
	std.dev.	0.0033	0.2409	0.0021	0.0028	0.0039	0.0047	0.0024
	rank	3	4	1	2	5	7	6
101085	mean	0.5916	0.5814	0.5914	0.6079	0.5845	0.5987	0.5864
	std.dev.	0.0549	0.0594	0.0597	0.0538	0.0455	0.0527	0.0395
	rank	3	7	4	1	6	2	5
147091	mean	0.5981	0.6270	0.6295	0.6353	0.6149	0.6626	0.6382
	std.dev.	0.0016	0.0573	0.0603	0.0607	0.0396	0.0733	0.0016
	rank	7	5	4	3	6	1	2
101087	mean	0.6419	0.6341	0.6356	0.6381	0.6360	0.6310	0.6405
	std.dev.	0.0051	0.0145	0.0091	0.0142	0.0100	0.0163	0.0085
	rank	1	6	5	3	4	7	2
253027	mean	0.7938	0.8103	0.8062	0.8055	0.7971	0.7917	0.8064
	std.dev.	0.0358	0.0315	0.0459	0.0377	0.0448	0.0552	0.0391
	rank	6	1	3	4	5	7	2
average rank		4.17	4.50	3.50	3.08	5.33	4.75	2.67
overall rank		4	5	3	2	7	6	1

**Table 9:** FSIM results for D=10.

Image		ME-DE	ME-FA	ME-BA	ME-MFO	ME-DA	ME-WOA	ME-GDEAR
Boats	mean	0.9521	0.9646	0.9585	0.9613	0.9548	0.9575	0.9664
	std.dev.	0.0157	0.0053	0.0111	0.0076	0.0119	0.0094	0.0063
	rank	7	2	4	3	6	5	1
Peppers	mean	0.7943	0.8648	0.6669	0.8619	0.8301	0.8250	0.8716
	std.dev.	0.1394	0.0064	0.1258	0.1083	0.1247	0.0164	0.0137
	rank	6	2	7	3	4	5	1
Goldhill	mean	0.8447	0.8778	0.8766	0.8211	0.8567	0.8258	0.8708
	std.dev.	0.0481	0.0328	0.0551	0.0468	0.0260	0.0615	0.0466
	rank	5	1	2	7	4	6	3
Lenna	mean	0.6295	0.6345	0.6253	0.6403	0.6409	0.6397	0.6465
	std.dev.	0.0902	0.0070	0.0235	0.1175	0.1346	0.0150	0.0281
	rank	6	5	7	3	2	4	1
House	mean	0.9525	0.9645	0.9994	0.9637	0.9484	0.8953	0.9600
	std.dev.	0.0131	0.0044	0.1913	0.0046	0.0110	0.1675	0.2078
	rank	5	2	1	3	6	7	4
12003	mean	0.5950	0.5990	0.5357	0.5919	0.5921	0.5820	0.5980
	std.dev.	0.1161	0.0112	0.0280	0.1845	0.1593	0.0163	0.0279
	rank	3	1	7	5	4	6	2
181079	mean	0.8247	0.8753	0.8441	0.8613	0.8572	0.8205	0.8788
	std.dev.	0.0950	0.0106	0.0953	0.0069	0.0192	0.0945	0.0053
	rank	6	2	5	3	4	7	1
175043	mean	0.9327	0.9551	0.9369	0.9486	0.9252	0.9322	0.9488
	std.dev.	0.0168	0.0070	0.1852	0.0045	0.0266	0.2206	0.0054
	rank	5	1	4	3	7	6	2
101085	mean	0.8335	0.8441	0.8773	0.8331	0.8345	0.8270	0.8381
	std.dev.	0.1426	0.0070	0.0455	0.1606	0.1413	0.0620	0.0501
	rank	5	2	1	6	4	7	3
147091	mean	0.8307	0.8958	0.8308	0.8848	0.8706	0.8642	0.8769
	std.dev.	0.1192	0.0067	0.0587	0.0541	0.0602	0.0833	0.0591
	rank	7	1	6	2	4	5	3
101087	mean	0.8122	0.8123	0.8261	0.8417	0.8915	0.8194	0.8376
	std.dev.	0.1178	0.0074	0.0082	0.1172	0.0180	0.1316	0.0149
	rank	7	6	4	2	1	5	3
253027	mean	0.9057	0.9173	0.9069	0.9125	0.8978	0.9079	0.9197
	std.dev.	0.0168	0.0106	0.0116	0.0069	0.0199	0.0114	0.0131
	rank	6	2	5	3	7	4	1
average rank		5.67	2.25	4.42	3.58	4.42	5.58	2.08
overall rank		7	2	4.5	3	4.5	6	1

**Table 10:** Dice score results for D=3.

Image		ME-DE	ME-BA	ME-ALO	ME-DA	ME-MVO	ME-WOA	ME-GDEAR
12003	mean	0.7775	0.7537	0.9412	0.7589	0.8207	0.8203	0.8128
	std.dev.	0.0634	0.0706	0.0000	0.0676	0.0000	0.0000	0.0662
	rank	5	7	1	6	2	3	4
181079	mean	0.3865	0.6533	0.7601	0.6533	0.7848	0.7847	0.6533
	std.dev.	0.0651	0.0000	0.0000	0.0000	0.0000	0.0000	0.0000
	rank	7	4	3	6	1	2	5
175043	Mean	0.8148	0.8421	0.8355	0.8416	0.8314	0.8537	0.9438
	std.dev.	0.0053	0.0521	0.0484	0.0523	0.0426	0.0578	0.0557
	rank	7	3	5	4	6	2	1
101085	mean	0.6533	0.9412	0.6533	0.8207	0.8203	0.6533	0.9412
	std.dev.	0.0000	0.0000	0.0000	0.0000	0.0000	0.0000	0.0000
	rank	5	1.5	6.5	3	4	6.5	1.5
147091	mean	0.7967	0.9412	0.7875	0.8224	0.8271	0.7615	0.9412
	std.dev.	0.0268	0.0000	0.0563	0.0058	0.0102	0.0579	0.0000
	rank	5	1.5	6	4	3	7	1.5
101087	mean	0.6533	0.9412	0.6533	0.8207	0.8203	0.6533	0.9412
	std.dev.	0.0000	0.0000	0.0000	0.0000	0.0000	0.0000	0.0000
	rank	5	1.5	6.5	3	4	6.5	1.5
253027	mean	0.8228	0.9412	0.7889	0.8225	0.8218	0.7966	0.9412
	std.dev.	0.0004	0.0000	0.0387	0.0008	0.0013	0.0389	0.0000
	rank	3	1.5	7	4	5	6	1.5
average rank		5.29	2.86	5.00	4.29	3.57	4.71	2.29
overall rank		7	2	6	4	3	5	1

**Table 11:** Dice score results for D=4.

Image		ME-DE	ME-BA	ME-ALO	ME-DA	ME-MVO	ME-WOA	ME-GDEAR
12003	mean	0.7749	0.7608	0.9394	0.7622	0.7934	0.8192	0.7031
	std.dev.	0.0539	0.0617	0.0000	0.0611	0.0197	0.0000	0.0893
	rank	4	6	1	5	3	2	7
181079	mean	0.4388	0.4603	0.7601	0.5376	0.6531	0.7847	0.5424
	std.dev.	0.0500	0.0326	0.0000	0.0024	0.0000	0.0000	0.0140
	rank	7	6	2	5	3	1	4
175043	mean	0.8226	0.8430	0.8329	0.8287	0.8442	0.8787	0.8383
	std.dev.	0.0157	0.0513	0.0421	0.0353	0.0524	0.0637	0.0471
	rank	7	3	5	6	2	1	4
101085	mean	0.5367	0.8196	0.5406	0.6531	0.8192	0.4894	0.9394
	std.dev.	0.0000	0.0000	0.0080	0.0000	0.0000	0.0403	0.0000
	rank	6	2	5	4	3	7	1
147091	mean	0.7885	0.8221	0.7607	0.7862	0.8251	0.7467	0.9394
	std.dev.	0.0311	0.0056	0.0635	0.0450	0.0081	0.0676	0.0000
	rank	4	3	6	5	2	7	1
101087	mean	0.5367	0.8196	0.5367	0.6531	0.8192	0.4496	0.9394
	std.dev.	0.0000	0.0000	0.0000	0.0000	0.0000	0.0000	0.0000
	rank	6	2	5	4	3	7	1
253027	mean	0.8133	0.8196	0.7710	0.8155	0.8192	0.7820	0.9394
	std.dev.	0.0029	0.0000	0.0351	0.0008	0.0000	0.0407	0.0000
	rank	5	2	7	4	3	6	1
average rank		5.57	3.43	4.43	4.71	2.71	4.43	2.71
overall rank		7	3	4.5	6	1.5	4.5	1.5

**Table 12:** Dice score results for D=5.

Image		ME-DE	ME-BA	ME-ALO	ME-DA	ME-MVO	ME-WOA	ME-GDEAR
12003	mean	0.7644	0.8196	0.9360	0.9360	0.7414	0.7268	0.9381
	std.dev.	0.0405	0.0000	0.0000	0.0000	0.0567	0.0615	0.0000
	rank	5	4	2.5	2.5	6	7	1
181079	mean	0.4826	0.7847	0.7597	0.7597	0.5485	0.6527	0.7589
	std.dev.	0.0401	0.0000	0.0000	0.0000	0.0247	0.0000	0.0000
	rank	7	1	2.5	2.5	6	5	4
175043	mean	0.8285	0.8266	0.8265	0.8398	0.8331	0.8672	0.8436
	std.dev.	0.0164	0.0491	0.0347	0.0459	0.0422	0.0665	0.0502
	rank	5	6	7	3	4	1	2
101085	mean	0.8196	0.9360	0.9360	0.5455	0.6527	0.8199	0.9381
	std.dev.	0.0000	0.0000	0.0000	0.0224	0.0000	0.0000	0.0000
	R	5	2.5	2.5	7	6	4	1
147091	mean	0.8233	0.9360	0.9360	0.7557	0.7748	0.8215	0.9381
	std.dev.	0.0049	0.0000	0.0000	0.0711	0.0455	0.0025	0.0000
	rank	4	2.5	2.5	7	6	5	1
101087	mean	0.8196	0.9360	0.9360	0.5368	0.6527	0.8199	0.9381
	std.dev.	0.0000	0.0000	0.0000	0.0000	0.0000	0.0000	0.0000
	rank	5	2.5	2.5	7	6	4	1
253027	mean	0.8196	0.9360	0.9360	0.7290	0.7342	0.8199	0.9381
	std.dev.	0.0000	0.0000	0.0000	0.0249	0.0285	0.0000	0.0000
	rank	5	2.5	2.5	7	6	4	1
average rank		5.14	3.00	3.14	5.14	5.71	4.29	1.57
overall rank		5.5	2	3	5.5	7	4	1

**Table 13:** Dice score results for D=10.

Image		ME-DE	ME-BA	ME-ALO	ME-DA	ME-MVO	ME-WOA	ME-GDEAR
12003	mean	0.6094	0.5869	0.5886	0.6294	0.5824	0.5020	0.6506
	std.dev.	0.0840	0.0210	0.1445	0.0711	0.0472	0.0769	0.0786
	rank	3	5	4	2	6	7	1
181079	mean	0.6346	0.6297	0.5256	0.6322	0.6273	0.7311	0.6383
	std.dev.	0.0147	0.0176	0.2312	0.0100	0.0147	0.0666	0.0274
	rank	3	5	7	4	6	1	2
175043	mean	0.8067	0.8171	0.7149	0.8165	0.8105	0.6004	0.7849
	std.dev.	0.0248	0.0173	0.1576	0.0136	0.0237	0.0864	0.0419
	rank	4	1	6	2	3	7	5
101085	mean	0.6779	0.5834	0.5608	0.6248	0.6573	0.6934	0.7201
	std.dev.	0.1409	0.0375	0.2307	0.1022	0.1205	0.2814	0.0711
	rank	3	6	7	5	4	2	1
147091	mean	0.5510	0.4469	0.6957	0.4718	0.4815	0.5239	0.6560
	std.dev.	0.1039	0.0235	0.0927	0.0452	0.0333	0.1151	0.0479
	rank	3	7	1	6	5	4	2
101087	mean	0.4310	0.4334	0.4041	0.4234	0.4646	0.3783	0.4421
	std.dev.	0.0333	0.0246	0.1592	0.0247	0.0062	0.0971	0.0677
	rank	4	3	6	5	1	7	2
253027	mean	0.5256	0.5191	0.5210	0.5157	0.5136	0.5233	0.5205
	std.dev.	0.0228	0.0268	0.0216	0.0215	0.0406	0.0238	0.0303
	rank	1	5	3	6	7	2	4
average rank		3.00	4.57	4.86	4.29	4.57	4.29	2.43
overall rank		2	4.5	7	3.5	4.5	3.5	1

**Table 14:** Results of Wilcoxon signed rank test.

	*p*-Value
ME-GDEAR vs. ME-DE	$5.8052\times 10^{-5}$
ME-GDEAR vs. ME-BA	$2.9061\times 10^{-6}$
ME-GDEAR vs. ME-GWO	$4.4433\times 10^{-9}$
ME-GDEAR vs. ME-DA	$9.4286\times 10^{-5}$
ME-GDEAR vs. ME-MVO	$1.1412\times 10^{-7}$
ME-GDEAR vs. ME-WOA	$3.6885\times 10^{-9}$

**Table 15:** Results of Friedman test.

Algorithm	Rank
ME-DE	4.24
ME-BA	3.92
ME-GWO	5.27
ME-DA	2.91
ME-MVO	4.90
ME-WOA	4.81
ME-GDEAR	1.96
*p*-value	$9.5625\times 10^{-17}$
chi-squared	87.6

## Data Availability

Data is contained within the article.
